# Optimal placebo-treatment comparisons in trials with an incomplete within-subject design and heterogeneous costs and variances

**DOI:** 10.1371/journal.pone.0283382

**Published:** 2023-04-20

**Authors:** Mirjam Moerbeek

**Affiliations:** Department of Methodology and Statistics, Utrecht University, Utrecht, The Netherlands; Mississippi State University, UNITED STATES

## Abstract

The aim of a clinical trial is to compare placebo to one or more treatments. The within-subject design is known to be more efficient than the between-subject design. However, in some trials that implement a within-subject design it is not possible to evaluate the placebo and all treatments within each subject. The design then becomes an incomplete within-subject design. An important question is how many subjects should be allocated to each combination of placebo and treatments. This paper studies optimal allocations of subjects in trials with a placebo and two treatments under heterogenous costs and variances. Two optimality criteria that consider the placebo-treatment contrasts simultaneously are considered, and the design is derived under a budgetary constraint. More subjects are allocated to those combinations with higher variances and lower costs. The optimal allocation is compared to the uniform allocation, which allocates equal number of subjects to each placebo and treatment combination, and to the complete within-subject design, where placebo and all treatments are available in each subject. The methodology is illustrated on the basis of an example on consultation time in primary care. A Shiny app is available to facilitate use of the methodology.

## Introduction

The randomized controlled trial is the gold standard for the comparison of multiple experimental conditions [1, 2]. An important question in the design phase of such a trial is how many subjects should be assigned to each of these conditions. Often a uniform design is chosen, so that an equal number of subjects is assigned to each condition. A rationale for the uniform design is that it is consistent with the view of clinical equipoise that must exist before the start of the trial [3]. However, the uniform design is not necessarily an efficient choice, especially so when costs and/or variances differ across the conditions [4]. A more efficient design is achieved when the conditions with highest variance and lowest costs are assigned more subjects.

The past twenty years various papers appeared on the optimal allocation of subjects to experimental conditions: for the comparison of two conditions on a quantitative outcome [5–7] or a categorical outcome [8–13] and for the comparison of more than two conditions on a quantitative outcome [3, 14–21] or a categorical outcome [22–24]. The main focus of the existing literature is on trials with a between-subject design, where each subject receives only one condition. Such a design is also referred to as the parallel group design. However, in some trials it is also possible to implement a within-subject design, so that each subject receives at least two conditions sequentially. Such a design is also referred to as a crossover design: subjects cross over from the one condition to the during the course of the trial [25, 26]. Because multiple conditions are available within each subject, each subject serves as its own control and hence the design is more efficient than the between-subject design.

In the ideal case each of the conditions is available within each subject so that the design is a complete within-subject design. However, in practice this is not always possible to achieve, especially so in trials with a limited study duration, a long onset time of treatment and/or a long wash-out period between subsequent conditions. Other reasons to limit the number of conditions per subject are participation burden and hence attrition, ethical concerns and the need to avoid test-retest effects. The latter occur when the same measurement instrument, such as a cognitive ability test or a behavioral test, is used too often in a limited amount of time. Consider as an example a pharmaceutical trial that compares different drugs to a placebo for children with attention deficit hyper activity disorder. Depending on the onset time of each drug, the number of different drugs than can be evaluated per child will be small in a trial of limited duration, especially so if washout periods are needed to avoid carry-over effects of a drug to the next period(s). In addition to that, too many drug switches per child may not be acceptable from an ethical point of view. Another example is a trial that compares the performance of different types of new and innovative sport shoes for professional marathon runners to a standard type. Runners are advised to limit the number of marathons per year and to take a few weeks or even months to recover before the next marathon. For that reason the number of type of sport shoes that can be evaluated per runner in a study of limited duration is rather small. In both examples the number of conditions per subject is lower than the actual number of conditions that is to be evaluated in the trail, hence the design is an incomplete within-subject design.

The aim of this contribution is to further study the optimal allocation of subjects for placebo-treatment comparisons under a budgetary constraint. The outcome is quantitative and the variances and costs are allowed to be heterogeneous. Attention is limited to trials with one placebo and two treatments. The optimal allocation of subjects for the incomplete within-subject design is compared to the uniform allocation. As such, the loss of efficiency that results from assigning equal number of subjects to each combination of experimental conditions can be quantified. Furthermore, the incomplete within-subject design is compared to the complete within-subject design. As such, the loss of efficiency from not having each experimental within each subject is quantified.

The optimal allocation of subjects to conditions depends on various factors: a budgetary constraint, the optimality criterion and the covariance matrix between the outcomes per subject. The budgetary constraint limits the number of subjects that can be included in the study because the costs of a study should not exceed its budget. Costs consist of, among others, overhead costs per subject and costs per subject per placebo and treatment. There exist various allocations of subjects for which the costs are less than or equal to the budget. An optimality criterion is therefore needed to select the optimal allocation. In this contribution the focus is on placebo-treatment comparisons. As there are two treatments, there are also two placebo-treatment comparisons. The allocation that is optimal for the comparison of placebo and treatment 1 may be far from optimal for the comparison of placebo to treatment 2. It is therefore necessary to use optimality criteria that consider both placebo-treatment comparisons simultaneously. The optimal allocation further depends on the variances and covariances between the outcomes within a subject. This contribution considers a general covariance matrix with heterogeneous variances and covariances. Once the costs and budget are specified, an a priori estimate of the covariance matrix is provided and the optimality criterion has been chosen, the optimal allocation of subjects can be derived. This paper builds upon previous research on optimal designs for the crossover trial with a budgetary constraint [27–29]. In these contributions two treatment conditions are taken into account and each subject receives both conditions. In the current paper three conditions are taken into account, but each subject receives only two out of these three conditions.

This contribution is organized as follows. The next section formulates the statistical model, shows how the means in the placebo and treatments are estimated along with the associated covariance matrix and introduces the optimal design methodology. Two optimality criteria are specified along with the budgetary constraint and it is explained how the optimal allocation to treatment combinations is derived numerically. The results section gives optimal allocations for the incomplete within-subject design and compares the optimal allocations to the uniform design and to the complete within-subject design on the basis of their efficiencies. An illustrative example on consultation time in primary care illustrates the use of the optimal design methodology. Conclusions, discussion and directions for future research are given in the final section.

## Methods

### Statistical model

In the within-subject design the data have a multilevel structure with multiple outcome measures nested within subjects. Such data can be analyzed using the multilevel model [30, 31], which is also known as the mixed model or hierarchical model [32].

We first focus on the model for the complete within-subject design, where each subject receives the placebo and both treatments. Subsequently it is shown how this model can be adjusted to account for the incomplete within-subject design. At the end of each treatment, outcome measurements are taken on each subject. The model for quantitative outcome *y*_*ij*_ for experimental condition *i* (*i* = 0 for placebo and *i* = 1, 2 for treatments 1 and 2, respectively) in subject *j* = 1,…*N* is given by

$$y_{ij}=\mu_{i}+e_{ij}$$ (1)

Here, *μ*_*i*_ is the mean score for condition *i*, and *e*_*ij*_ is the residual. For the latter we assume $E(e_{ij})=0,\;var(e_{ij})=\sigma_{i}^{2}$ and $cov(e_{ij},e_{i\prime j})=\sigma_{ii\prime }$. As such, the model allows for heterogenous variances and covariances. It should be noted that this model does not account for temporal effects, such as models for crossover trials often do.

The model can be written in matrix-vector notation:

$$y_{ij}=X_{j}\mu +Z_{j}e_{j}.$$ (2)

The vector of outcomes of subject *j* is

$$y_{j}={\left(\begin{array}{ccc} y_{0j} & y_{1j} & y_{2j} \end{array}\right)}^{T}.$$ (3)

The vector of placebo and treatment means is

$$\mu ={\left(\begin{array}{ccc} \mu_{0} & \mu_{1} & \mu_{2} \end{array}\right)}^{T},$$ (4)

with corresponding design matrix

$$X_{j}=(\begin{array}{ccc} 1 & 0 & 0\\ 0 & 1 & 0\\ 0 & 0 & 1 \end{array}).$$ (5)

In the complete within-subject design the design matrix is the identity matrix. The vector of residuals is of subject *j* is:

$$e_{j}={\left(\begin{array}{ccc} e_{0j} & e_{1j} & e_{2j} \end{array}\right)}^{T},$$ (6)

with corresponding design matrix

$$Z_{j}=(\begin{array}{ccc} 1 & 0 & 0\\ 0 & 1 & 0\\ 0 & 0 & 1 \end{array}).$$ (7)

Again the design matrix is the identity matrix in the complete within-subject design. The 3×3 covariance matrix of the residuals *e*_*j*_ is

$$D=(\begin{array}{ccc} \sigma_{0}^{2} & \sigma_{01} & \sigma_{02}\\ \sigma_{01} & \sigma_{1}^{2} & \sigma_{12}\\ \sigma_{02} & \sigma_{12} & \sigma_{2}^{2} \end{array}).$$ (8)

The covariance matrix of the response vector ***y***_*j*_ is calculated as

$$V_{j}=Z_{j}DZ_{j}^{T}.$$ (9)

The vector of placebo and treatment means is estimated as

$$\hat{\mu }={\left(\sum_{j=1}^{N}X_{j}^{T}V_{j}^{-1}X_{j}\right)}^{-1}\sum_{j=1}^{N}X_{j}^{T}V_{j}^{-1}y_{j},$$ (10)

with corresponding covariance matrix

$$var(\hat{\mu })={\left(\sum_{j=1}^{N}X_{j}^{T}V_{j}^{-1}X_{j}\right)}^{-1}.$$ (11)

In the incomplete within-subject design certain entries should be removed from the vectors with outcomes and residuals and certain rows should be removed from the design matrices. If the placebo is not available within subject *j*, then the first entry should be removed from the vectors *y*_*j*_ and *e*_*j*_ and the first row should be removed from the design matrices ***X***_*j*_ and ***Z***_*j*_. If treatment *i* is not available within subject *j*, then entry *i*+1 should be removed from the vectors *y*_*j*_ and *e*_*j*_ and row *i*+1 should be removed from the design matrices ***X***_*j*_ and ***Z***_*j*_. Eqs (9)–(11) can then be used to estimate the placebo and treatment means and the associated covariance matrix.

### Optimal design methodology

#### Optimality criteria

To derive the optimal allocation of subjects to treatment combinations an optimality criterion is needed. The focus is on placebo-treatment comparisons, of which there are two in a trial with two treatments. For both of them a contrast $a_{0i}^{T}\mu$ can be formulated, with $a_{01}^{T}=(1,-1,0)$ and $a_{02}^{T}=(1,0,-1)$. The variance of these contrasts is calculated as

$$\Phi_{0i}=var(a_{0i}^{T}\hat{\mu })=a_{0i}^{T}var(\hat{\mu })a_{0i}.$$ (12)

The optimal allocation is the one that minimizes the contrast variance Φ_0*i*_. The allocation that is optimal for the first contrast may be far from optimal for the second. For that reason the contrast variances Φ_0*i*_ should be considered simultaneously. This will be done by implementing a *D*_*A*_-optimal design [33, 34] or a compound optimal design [35].

For the *D*_*A*_-optimal design both vectors of contrast coefficients are stacked in one matrix:

$$A^{T}=(\begin{array}{ccc} 1 & -1 & 0\\ 1 & 0 & -1 \end{array}).$$ (13)

The optimality criterion to be minimized is then

$$\Phi_{D_{A}}=\det\left(A^{T}var(\hat{\mu })A\right).$$ (14)

As such, the optimal allocation minimizes the volume of the confidence ellipsoid of the contrasts.

For the compound optimal design a linear combination of the contrast variances Φ_0*i*_ is minimized:

$$\Phi_{compound}=\lambda_{1}\Phi_{01}+(1-\lambda_{1})\Phi_{02},$$ (15)

where 0≤*λ*_1_≤1. The user-specified weight *λ*_1_ reflects the relative importance of the first contrast in the compound optimal design. This criterion was used previously in a between-subject design where costs and variances were equal across placebo and treatments [14].

The optimal design may be compared to other designs that are of interest, for instance the uniform design. This may be done by means of the relative efficiency. For the *D*_*A*_-optimal design the relative efficiency of an allocation *ξ* versus the optimal allocation ξ* is

$${RE}_{D_{A}}=\frac{{{[\Phi }_{D_{A}}(\xi^{*})]}^{½}}{[{\Phi_{D_{A}}(\xi )]}^{½}}.$$ (16)

The parts in square brackets of the numerator and denominator are the value of the optimality criterion $\Phi_{D_{A}}$ as found for the allocations ξ* and *ξ*, respectively. Note that both the numerator and denominator are taken to the power ½, since two contrasts are taken into account. For the compound optimal design the relative efficiency is calculated as

$${RE}_{compound}=\frac{\Phi_{compound}(\xi^{*})}{\Phi_{compound}(\xi )}.$$ (17)

The numerator and denominator are the value of the optimality criterion Φ_*compound*_ as found for the allocations ξ* and *ξ*, respectively.

The relative efficiency lies between 0 and 1 and its reciprocal quantifies how many times a trial with allocation *ξ* should be replicated to perform as well as the optimal allocation ξ*. A trial with *RE* = 0.8 need to be replicated 1.25 times and a trail with *RE* = 0.9 need to be replicated 1.11 times. Stated differently, a trail with *RE* = 0.8 needs an increase in sample size of $\left(\frac{1}{0.8}-1\right)\times 100\%=25\%$ and a trial with *RE* = 0.9 needs an increase in sample size of $\left(\frac{1}{0.9}-1\right)\times 100\%=11\%$.

#### Budgetary constraint

As is obvious, any design becomes more efficient when sample size increases. In practice, however, sample size cannot increase within bounds due to financial limitations. A budgetary constraint is therefore used to derive the optimal allocation to treatment combinations.

The overhead costs per subject are denoted *C*_*s*_ and consist of costs such as administrative costs, incentives and insurances. These costs do not depend on the number and order of the conditions that are assigned to a subject. The costs for placebo per subject are denoted *C*_0_ and the costs for treatment *i* per subject are denoted *C*_*i*_. The budget that is available is denoted *B*.

The costs per subject depend on which condition(s) the subject receives. In the incomplete within-subject design the costs per subject are *C*_*s*01_ = *C*_*s*_+*C*_0_+*C*_1_ for a subject who receives placebo combined with treatment 1, *C*_*s*02_ = *C*_*s*_+*C*_0_+*C*_2_ for a subject who receives placebo combined with treatment 2 and *C*_*s*12_ = *C*_*s*_+*C*_1_+*C*_2_ for a subject who receives treatment 1 combined with treatment 2. The corresponding numbers of subjects are denoted *N*_01_, *N*_02_ and *N*_12_, respectively. The costs are then *C*_*s*01_*N*_01_+*C*_*s*02_*N*_02_+*C*_*s*12_*N*_12_. The design space is Ω = (*N*_01_≥0, *N*_02_≥0, *N*_12_≥0|*costs*≤*B*), with *N*_01_, *N*_02_ and *N*_12_ integers. The optimal allocation ξ* is the one for which the optimality criterion $\Phi_{D_{A}}$ or Φ_*compound*_ is minimized.

In the complete within-subject design the costs per subject are *C*_*s*012_ = *C*_*s*_+*C*_0_+*C*_1_+*C*_2_. The number of subjects that can be included are *N*_012_ = *B*/*C*_*s*012_. As all subjects receive placebo combined with treatments 1 and 2, it is not necessary to derive the optimal allocation of subjects.

#### Derivation of optimal allocation

Simple mathematical expressions for the optimal allocation cannot be derived. For that reason the optimal allocation is found numerically. For optimization problems like this it is straightforward to use an optimizer for functions with constraints, such as the optimizer constrOptim.nl in the R [36] package alabama [37]. In this optimization problem the function is the variance of the treatment effect estimator (with the sample sizes as parameters), and the constraints are the cost constraint and the constraint that all sample sizes are natural numbers (i.e. integers of zero or larger). Unfortunately, the optimizer constrOptim.nl appeared to be very sensitive to the starting values of the iterative optimization process. Also, the solution by default produced non-integer sample sizes. For that reason another approach to find the optimal allocation was used: the variance of the treatment effect estimator is calculated for all possible combinations of integer samples for the which the cost constraint holds. The combination that results in the smallest variance is the optimal allocation. The amount of time this approach requires depends on the number of combinations of sample sizes, which in its turn depends on the costs and budget. For instance, the optimal allocations in Table 1 in the next section took a few seconds each to calculate, and those in Table 2 took about one minute each to calculate using a computer with 16 GB RAM and a processor with 2.8 GHz.

**Table 1:** Optimal sample sizes for the incomplete within–subject design with scenario 1.

	*C*_0_ = 10, *C*_1_ = 20, *C*_2_ = 20			*C*_0_ = 10, *C*_1_ = 20, *C*_2_ = 30			*C*_0_ = 10, *C*_1_ = 20, *C*_2_ = 40		
variances	$N_{01}^{*}$	$N_{02}^{*}$	N*	$N_{01}^{*}$	$N_{02}^{*}$	N*	$N_{01}^{*}$	$N_{02}^{*}$	N*
	compound optimal design with *λ*_1_ = 0.25								
$\sigma_{0}^{2}=1,\sigma_{1}^{2}=1,\sigma_{2}^{2}=1$	141	243	384	138	229	367	133	218	351
$\sigma_{0}^{2}=1,\sigma_{1}^{2}=2,\sigma_{2}^{2}=3$	123	261	384	124	242	366	118	231	349
$\sigma_{0}^{2}=1,\sigma_{1}^{2}=2,\sigma_{2}^{2}=4$	111	273	384	110	255	365	103	244	347
	compound optimal design with *λ*_1_ = 0.5								
$\sigma_{0}^{2}=1,\sigma_{1}^{2}=1,\sigma_{2}^{2}=1$	192	192	384	194	177	371	185	173	358
$\sigma_{0}^{2}=1,\sigma_{1}^{2}=2,\sigma_{2}^{2}=3$	173	211	384	166	203	369	170	186	356
$\sigma_{0}^{2}=1,\sigma_{1}^{2}=2,\sigma_{2}^{2}=4$	159	225	384	152	216	368	155	199	354
	compound optimal design with *λ*_1_ = 0.75								
$\sigma_{0}^{2}=1,\sigma_{1}^{2}=1,\sigma_{2}^{2}=1$	243	141	384	237	137	374	238	127	365
$\sigma_{0}^{2}=1,\sigma_{1}^{2}=2,\sigma_{2}^{2}=3$	225	159	384	222	151	373	215	147	362
$\sigma_{0}^{2}=1,\sigma_{1}^{2}=2,\sigma_{2}^{2}=4$	211	173	384	208	164	372	208	153	361
	*D*_*A*_-optimal design								
$\sigma_{0}^{2}=1,\sigma_{1}^{2}=1,\sigma_{2}^{2}=1$	192	192	384	194	177	371	193	166	359
$\sigma_{0}^{2}=1,\sigma_{1}^{2}=2,\sigma_{2}^{2}=3$	190	194	384	194	177	371	193	166	359
$\sigma_{0}^{2}=1,\sigma_{1}^{2}=2,\sigma_{2}^{2}=4$	189	195	384	194	177	371	185	173	358

Note: *B* = 50,000 and *C*_*s*_ = 100 and *ρ*_*ii*′_ = 0.5

Subjects receive treatment combinations placebo with treatment 1 or placebo with treatment2.

**Table 2:** Optimal sample sizes for the incomplete within–subject design with scenario 2.

	*C*_0_ = 10, *C*_1_ = 20, *C*_2_ = 20				*C*_0_ = 10, *C*_1_ = 20, *C*_2_ = 30				*C*_0_ = 10, *C*_1_ = 20, *C*_2_ = 40			
variances	$N_{01}^{*}$	$N_{02}^{*}$	$N_{12}^{*}$	N*	$N_{01}^{*}$	$N_{02}^{*}$	$N_{12}^{*}$	N*	$N_{01}^{*}$	$N_{02}^{*}$	$N_{12}^{*}$	N*
	compound optimal design with *λ*_1_ = 0.25											
$\sigma_{0}^{2}=1,\sigma_{1}^{2}=1,\sigma_{2}^{2}=1$	119	229	34	382	116	213	34	363	117	201	29	347
$\sigma_{0}^{2}=1,\sigma_{1}^{2}=2,\sigma_{2}^{2}=3$	54	210	112	376	57	196	101	354	64	184	88	336
$\sigma_{0}^{2}=1,\sigma_{1}^{2}=2,\sigma_{2}^{2}=4$	34	216	125	375	39	202	111	352	41	189	102	332
	compound optimal design with *λ*_1_ = 0.5											
$\sigma_{0}^{2}=1,\sigma_{1}^{2}=1,\sigma_{2}^{2}=1$	167	167	47	381	172	161	34	367	171	151	32	354
$\sigma_{0}^{2}=1,\sigma_{1}^{2}=2,\sigma_{2}^{2}=3$	111	153	112	376	113	144	101	358	113	133	96	342
$\sigma_{0}^{2}=1,\sigma_{1}^{2}=2,\sigma_{2}^{2}=4$	89	161	125	375	94	152	110	356	94	142	103	339
	compound optimal design with *λ*_1_ = 0.75											
$\sigma_{0}^{2}=1,\sigma_{1}^{2}=1,\sigma_{2}^{2}=1$	229	119	34	382	227	111	33	371	224	104	33	361
$\sigma_{0}^{2}=1,\sigma_{1}^{2}=2,\sigma_{2}^{2}=3$	178	100	99	377	177	91	95	363	177	85	89	351
$\sigma_{0}^{2}=1,\sigma_{1}^{2}=2,\sigma_{2}^{2}=4$	157	107	112	376	158	99	104	361	158	94	96	348
	*D*_*A*_-optimal design											
$\sigma_{0}^{2}=1,\sigma_{1}^{2}=1,\sigma_{2}^{2}=1$	132	132	112	376	140	120	100	360	147	111	89	347
$\sigma_{0}^{2}=1,\sigma_{1}^{2}=2,\sigma_{2}^{2}=3$	99	109	164	372	107	96	151	354	118	86	136	340
$\sigma_{0}^{2}=1,\sigma_{1}^{2}=2,\sigma_{2}^{2}=4$	98	110	164	372	108	94	152	354	113	85	141	339

Note: *B* = 50,000 and *C*_*s*_ = 100 and *ρ*_*ii*′_ = 0.5

Subjects receive treatment combinations placebo with treatment 1, placebo with treatment2 or treatment 1 with treatment 2.

To facilitate the use of the optimal design methodology in this contribution, a Shiny app was developed. It can be assessed online at https://utrecht-university.shinyapps.io/placebo_treatment/.

## Results

The following subsections present optimal allocations for three different combinations of costs per subject for placebo and treatments 1 and 2. The first is *C*_0_ = 10, *C*_1_ = 20, *C*_2_ = 20 so that the costs of both treatments are equal to one another and twice as large as those for placebo. The second is *C*_0_ = 10, *C*_1_ = 20, *C*_2_ = 30 so that the costs of treatment 1 are twice as large as those of placebo and those of treatment 2 are one and a half times as large as those of treatment 1. The third is *C*_0_ = 10, *C*_1_ = 20, *C*_2_ = 40 so that the costs of treatment 1 are twice as large as those of placebo and those of treatment 2 are twice as large as those of treatment 1. The overhead costs per subject are *C*_*S*_ = 100 and the budget is *B* = 50000.

Furthermore, three different covariance matrices ***D*** of the residuals are considered. For the first we have $\sigma_{0}^{2}=1,\sigma_{1}^{2}=1,\sigma_{2}^{2}=1$. The variances are equal for placebo and both treatments. For the second we have $\sigma_{0}^{2}=1,\sigma_{1}^{2}=2,\sigma_{2}^{2}=3$. The variances in treatments 1 and 2 are twice and thrice as large as the variance in placebo, respectively. For the third we have $\sigma_{0}^{2}=1,\sigma_{1}^{2}=2,\sigma_{2}^{2}=4$. Here the variance in treatments 1 and 2 are twice and four times as large as in placebo, respectively. For the complete and incomplete within-subject design all pairwise correlations are set to *ρ*_*ii*′_ = 0.5, so that they are of large size according to Cohen [38].

For each combination of costs and covariance matrix the *D*_*A*_-optimal design and compound optimal design are derived. For the latter three different values of the weight *λ*_1_ are used: *λ*_1_ = 0.25, 0.5 and 0.75. For the first value of *λ*_1_ the main focus is on the comparison of placebo with treatment 2, for the second both placebo-treatment comparisons are of equal importance and for the third the main focus is on the comparison of placebo with treatment 1.

### Incomplete within-subject design

For this design there are two conditions per subject. Two different scenarios are considered. In scenario 1 subjects are allocated to receive either placebo with treatment 1 or placebo with treatment 2. In scenario 2 subjects are allocated to receive either placebo with treatment 1, placebo with treatment 2, or treatment 1 with treatment 2. In scenario 1 the combination of both treatments 1 and 2 is not taken into account. This scenario may be chosen when it is considered unethical to allocate both treatments to a subject, for instance when both are expected to have harmful side effects.

Table 1 presents the optimal sample sizes $N_{01}^{*},N_{02}^{*}$ and their sum N* for scenario 1. We first discuss results for the compound optimal designs. For any combination of costs *C*_0_, *C*_1_, *C*_2_ and weight *λ*_1_ the optimal sample size $N_{01}^{*}$ decreases, and hence the optimal sample size $N_{02}^{*}$ increases, when the ratio $\sigma_{2}^{2}/\sigma_{1}^{2}$ becomes larger. Fewer subjects receive placebo combined with treatment 1, and hence more receive placebo combined with treatment 2, if the variance of outcomes in treatment 2 increases relative to the variance of the outcomes in treatment 1. This confirms results previously found for a between-subject comparison of two means: more subjects are assigned to the treatment with highest variance.

For those designs with equal costs *C*_1_ = *C*_2_ = 20 in treatments 1 and 2, the total sample size remains constant at N* = 384, irrespective of the chosen variances and weight *λ*_1_. This can be explained as follows: as both treatment combinations are equally expensive, a decrease of the optimal sample size $N_{01}^{*}$ by a certain amount results in an increase of the optimal sample size $N_{02}^{*}$ by the same amount. For unequal costs (*C*_1_, *C*_2_) = (20,30) and (20,40) this result does not hold: increasing the ratio $\sigma_{2}^{2}/\sigma_{1}^{2}$ results in lower $N_{01}^{*}$, higher $N_{02}^{*}$ but lower N*.

For any combination of variance ratio $\sigma_{2}^{2}/\sigma_{1}^{2}$ and weight *λ*_1_ the optimal sample size $N_{02}^{*}$ and total sample size N* decrease if the costs *C*_2_ for treatment 2 increase. Fewer subjects are assigned to placebo combined with treatment 2 if the costs for treatment 2 increase and consequently the total sample size decreases as well. The relationship between *C*_2_ and $N_{01}^{*}$ is not always monotone.

The optimal sample sizes further depend on the weight *λ*_1_ of the compound optimal design. For any combination of costs *C*_0_, *C*_1_, *C*_2_ and variance ratio $\sigma_{2}^{2}/\sigma_{1}^{2}$ the optimal sample size $N_{01}^{*}$ increases, and hence the optimal sample size $N_{02}^{*}$ decreases, if the weight *λ*_1_ increases. This is also obvious: when more emphasis is put on the comparison of placebo to treatment 1 rather than to treatment 2, more subjects will be allocated to receive placebo combined with treatment 1 than placebo combined with treatment 2.

Only when variances and costs are equal for treatments 1 and 2 and when there is equal interest in both placebo-treatment comparisons (i.e. *λ*_1_ = 0.5) the optimal allocation is the uniform allocation. In all other cases the two optimal sample sizes $N_{01}^{*}$ and $N_{02}^{*}$ are different from each other. In the most extreme cases in Table 1 $N_{02}^{*}=0.5N_{01}^{*}$ and $N_{02}^{*}=2.5N_{01}^{*}$.

We now focus on results for the *D*_*A*_-optimal design. The optimal sample sizes $N_{01}^{*},N_{02}^{*}$ and their sum N* are not at all or only slightly influenced by the variance ratio $\sigma_{2}^{2}/\sigma_{1}^{2}$. Increasing the costs *C*_2_ of treatment 2 hardly influences the optimal sample size $N_{01}^{*}$, but results in a decrease of the optimal sample size $N_{02}^{*}$ and total sample size N*. Fewer subjects receive placebo combined with treatment 2 if the costs for treatment 2 increase. As a result of that, total sample size also becomes smaller.

Table 2 presents the optimal sample sizes $N_{01}^{*},N_{02}^{*},N_{12}^{*}$ and their sum N* for scenario 2. Again, results for the compound optimal designs are discussed first. For any combination of costs *C*_0_, *C*_1_, *C*_2_ and weight *λ*_1_ it can be observed that increasing the variance ratio $\sigma_{2}^{2}/\sigma_{1}^{2}$ results in decreasing optimal sample size $N_{01}^{*}$, decreasing and subsequently increasing optimal sample size $N_{02}^{*}$, increasing optimal sample size $N_{12}^{*}$ and decreasing total sample size N*. The effects of $\sigma_{2}^{2}/\sigma_{1}^{2}$ on $N_{02}^{*}$ and N* are not as pronounced as those on $N_{01}^{*}$ and $N_{12}^{*}$.

For any combination of variance ratio $\sigma_{2}^{2}/\sigma_{1}^{2}$ and weight *λ*_1_ the optimal sample sizes $N_{02}^{*},N_{12}^{*}$ and total sample size N* decrease with increasing costs *C*_2_. As is obvious, fewer subjects are assigned to receive treatment 2, either combined with placebo or treatment 1, if the costs of treatment 2 increase. The relationship between $N_{01}^{*}$ and $\sigma_{2}^{2}/\sigma_{1}^{2}$ is not always monotone.

For any combination of costs *C*_0_, *C*_1_, *C*_2_ and variance ratio $\sigma_{2}^{2}/\sigma_{1}^{2}$ the optimal sample size $N_{01}^{*}$ increases while the optimal sample size $N_{02}^{*}$ decreases if the weight *λ*_1_ increases. This is obvious since more subjects are assigned to receive placebo combined with treatment 1 rather than placebo combined with treatment 2 if comparing placebo to treatment 1 becomes of more interest. The relation between *λ*_1_ and $N_{12}^{*}$ is not always monotone.

We now focus on the optimal allocation for the *D*_*A*_-optimality criterion. The optimal sample sizes $N_{01}^{*},N_{02}^{*}$ and total sample size N* decrease with increasing variance ratio $\sigma_{2}^{2}/\sigma_{1}^{2}$, whereas the optimal sample size $N_{12}^{*}$ increases. Furthermore, the optimal sample size $N_{01}^{*}$ increases with increasing costs *C*_2_ whereas the optimal sample sizes $N_{02}^{*}$ and $N_{12}^{*}$ and total sample size N* decrease.

### Complete within-subject design

For this design all subjects receive placebo combined with treatments 1 and 2 and it is therefore not necessary to derive an optimal allocation. As was already explained, the number of subjects is *N*_012_ = *B*/*C*_*s*012_. In other words: the number of subjects is as large as the budget allows. It can be calculated that *N*_012_ = 333 subjects can be included when *C*_0_ = 10, *C*_1_ = 20, *C*_2_ = 20, *N*_012_ = 312 when *C*_0_ = 10, *C*_1_ = 20, *C*_2_ = 30 and *N*_012_ = 294 when *C*_0_ = 10, *C*_1_ = 20, *C*_2_ = 30.

### Efficiency of uniform allocation

Table 3 presents the efficiency of the uniform allocation relative to the optimal one. These efficiencies are equal or almost equal to 1 for the *D*_*A*_-optimality criterion, so the uniform design performs extremely well for this criterion. For the compound optimal designs with λ_1_ = 0.5 all relative efficiencies are above 0.9. In the case of equal interest in both placebo-treatment comparisons the uniform design is highly efficient. For the compound optimal designs with *λ*_1_ = 0.75 only three out of the 18 relative efficiencies are below 0.9. The lowest one is 0.84, meaning the uniform allocation needs an increase in total sample size of 20% to perform as well as the optimal allocation. For the compound optimal designs with *λ*_1_ = 0.25 11 out of 18 of the relative efficiencies are below 0.9 but all of them are still above 0.85.

**Table 3:** Efficiency of uniform allocation relative to optimal allocation.

	*C*_0_ = 10, *C*_1_ = 20, *C*_2_ = 20		*C*_0_ = 10, *C*_1_ = 20, *C*_2_ = 30		*C*_0_ = 10, *C*_1_ = 20, *C*_2_ = 40	
variances	*RE*_*W*1_	*RE*_*W*2_	*RE*_*W*1_	*RE*_*W*2_	*RE*_*W*1_	*RE*_*W*2_
	compound optimal design with *λ*_1_ = 0.25					
$\sigma_{0}^{2}=1,\sigma_{1}^{2}=1,\sigma_{2}^{2}=1$	0.943	0.877	0.951	0.878	0.957	0.875
$\sigma_{0}^{2}=1,\sigma_{1}^{2}=2,\sigma_{2}^{2}=3$	0.887	0.900	0.898	0.911	0.906	0.916
$\sigma_{0}^{2}=1,\sigma_{1}^{2}=2,\sigma_{2}^{2}=4$	0.851	0.871	0.863	0.885	0.873	0.893
	compound optimal design with *λ*_1_ = 0.5					
$\sigma_{0}^{2}=1,\sigma_{1}^{2}=1,\sigma_{2}^{2}=1$	1.000	0.928	0.999	0.920	0.995	0.908
$\sigma_{0}^{2}=1,\sigma_{1}^{2}=2,\sigma_{2}^{2}=3$	0.990	0.991	0.993	0.993	0.993	0.988
$\sigma_{0}^{2}=1,\sigma_{1}^{2}=2,\sigma_{2}^{2}=4$	0.972	0.978	0.977	0.984	0.979	0.983
	compound optimal design with *λ*_1_ = 0.75					
$\sigma_{0}^{2}=1,\sigma_{1}^{2}=1,\sigma_{2}^{2}=1$	0.943	0.877	0.933	0.860	0.920	0.840
$\sigma_{0}^{2}=1,\sigma_{1}^{2}=2,\sigma_{2}^{2}=3$	0.972	0.969	0.965	0.958	0.956	0.944
$\sigma_{0}^{2}=1,\sigma_{1}^{2}=2,\sigma_{2}^{2}=4$	0.99	0.987	0.985	0.981	0.979	0.968
	*D*_*A*_-optimal design					
$\sigma_{0}^{2}=1,\sigma_{1}^{2}=1,\sigma_{2}^{2}=1$	1.000	0.999	0.998	0.996	0.994	0.986
$\sigma_{0}^{2}=1,\sigma_{1}^{2}=2,\sigma_{2}^{2}=3$	1.000	0.990	0.999	0.992	0.995	0.987
$\sigma_{0}^{2}=1,\sigma_{1}^{2}=2,\sigma_{2}^{2}=4$	1.000	0.987	0.999	0.990	0.995	0.986

Note: *B* = 50,000 and *C*_*s*_ = 100 and *ρ*_*ii*′_ = 0.5.

*RE*_*W*1_ relative efficiency of uniform versus optimal allocation for the within–subject design with scenario 1.

*RE*_*W*2_ relative efficiency of uniform versus optimal allocation for the within–subject design with scenario 2.

### Efficiency of the incomplete within-subject design

A comparison of the total sample sizes in Tables 1 and 2 and those calculated for the complete within-subject design show that the total sample size is smallest for the complete-within subject design, followed by the incomplete within-subject design with scenario 1, and the incomplete within-subject design with scenario 2. The fewer experimental conditions are available within a subject, the more subjects can be included in the trial. However, a larger number of subjects does not imply a larger design efficiency. This follows from Table 4, which presents the efficiency of the incomplete within-subject designs relative to the complete within-subject design. The complete within-subject design is the most efficient, followed by the incomplete within-subject design with scenario 2 and with scenario 1, respectively. The more experimental conditions are available within a subject, the more efficient the design. Allowing for the combination of treatment 1 with 2 results in a more efficient design.

**Table 4:** Efficiency of the incomplete within–subject design relative to the complete within–subject design.

	*C*_0_ = 10, *C*_1_ = 20, *C*_2_ = 20		*C*_0_ = 10, *C*_1_ = 20, *C*_2_ = 30		*C*_0_ = 10, *C*_1_ = 20, *C*_2_ = 40	
variances	*RE*_*W*1_	*RE*_*W*2_	*RE*_*W*1_	*RE*_*W*2_	*RE*_*W*1_	*RE*_*W*2_
	compound optimal design with *λ*_1_ = 0.25					
$\sigma_{0}^{2}=1,\sigma_{1}^{2}=1,\sigma_{2}^{2}=1$	0.699	0.713	0.713	0.724	0.723	0.732
$\sigma_{0}^{2}=1,\sigma_{1}^{2}=2,\sigma_{2}^{2}=3$	0.656	0.734	0.666	0.736	0.674	0.738
$\sigma_{0}^{2}=1,\sigma_{1}^{2}=2,\sigma_{2}^{2}=4$	0.681	0.776	0.690	0.776	0.697	0.776
	compound optimal design with *λ*_1_ = 0.5					
$\sigma_{0}^{2}=1,\sigma_{1}^{2}=1,\sigma_{2}^{2}=1$	0.659	0.674	0.678	0.691	0.695	0.706
$\sigma_{0}^{2}=1,\sigma_{1}^{2}=2,\sigma_{2}^{2}=3$	0.590	0.662	0.605	0.671	0.618	0.679
$\sigma_{0}^{2}=1,\sigma_{1}^{2}=2,\sigma_{2}^{2}=4$	0.599	0.683	0.613	0.690	0.624	0.696
	compound optimal design with *λ*_1_ = 0.75					
$\sigma_{0}^{2}=1,\sigma_{1}^{2}=1,\sigma_{2}^{2}=1$	0.699	0.713	0.727	0.739	0.752	0.763
$\sigma_{0}^{2}=1,\sigma_{1}^{2}=2,\sigma_{2}^{2}=3$	0.605	0.671	0.627	0.689	0.646	0.705
$\sigma_{0}^{2}=1,\sigma_{1}^{2}=2,\sigma_{2}^{2}=4$	0.592	0.666	0.612	0.682	0.629	0.695
	*D*_*A*_-optimal design					
$\sigma_{0}^{2}=1,\sigma_{1}^{2}=1,\sigma_{2}^{2}=1$	0.577	0.627	0.594	0.639	0.609	0.650
$\sigma_{0}^{2}=1,\sigma_{1}^{2}=2,\sigma_{2}^{2}=3$	0.550	0.654	0.566	0.663	0.580	0.671
$\sigma_{0}^{2}=1,\sigma_{1}^{2}=2,\sigma_{2}^{2}=4$	0.553	0.664	0.569	0.673	0.583	0.681

Note: *B* = 50,000 and *C*_*s*_ = 100 and *ρ*_*ii*′_ = 0.5.

*RE*_*W*1_ relative efficiency of the incomplete within–subject design with scenario 1 versus the complete within–subject design.

*RE*_*W*2_ relative efficiency of the incomplete within–subject design with scenario 2 versus the complete within–subject design.

### Illustrative example: Consultation time in primary care

The average consultation time in primary care in the Netherlands is only 10 minutes [39]. This short amount of time may result in too high work pressure, dissatisfaction, stress and burnout of the general practitioner. Suppose a trial is set up to study the effects of longer consultation time. There are three experimental conditions: the standard consultation time of 10 minutes, consultation time of 15 minutes and consultation time of 20 minutes. The time period of data collection is limited so that only two conditions can be evaluated per general practitioner, and one week is available per condition. Furthermore, each general practitioner should evaluate the standard consultation time. This results in two combinations of consultation time: combination 1 has 10 minutes combined with 15 minutes, and combination 2 has 10 minutes combined with 20 minutes.

The costs of a consult are 10.59 euros per ten minutes. Suppose a general practitioner spends 20 hours per week on consults. Then he or she can do 120 consults of 10 minutes a week, 80 consults of 15 minutes a week and 60 consults of 20 minutes a week. To compensate for the decrease in the number of consults that can be done per week, general practitioners in combination 1 receive *C*_1_ = 40*10.59 = 423.6 euros and those in combination 2 receive *C*_2_ = 60*10.59 = 635.4 euros to hire a colleague. On top of that, they all receive incentives of *C*_*s*_ = 500 euros per practitioner. Furthermore, *C*_0_ = 0, meaning that no compensation is given for the week in which the standard consultation time is used. From these costs is follows *C*_*s*01_ = 923.6 and *C*_*s*02_ = 1135.4. The budget is *B* = 100,000 euros.

To derive the optimal allocation a priori estimates of the variances and covariances are needed. The outcome variable is stress level, which is measured at the end of the two weeks using an instrument that ranges from 0 to 100. Relevant a priori estimates of the variances and covariances were unfortunately not available in the literature. In such a case one could consult an expert to get plausible estimates. Let us assume the anticipated variances are $\sigma_{0}^{2}=100$ for 10 minutes consultation time, $\sigma_{1}^{2}=125$ for 15 minutes and $\sigma_{2}^{2}=150$ for 20 minutes. The correlations are anticipated to be *ρ*_*ii*′_ = 0.3 so that the covariances are *σ*_01_ = 33.5, *σ*_02_ = 36.7 and *σ*_12_ = 41.1.

**Fig 1:**
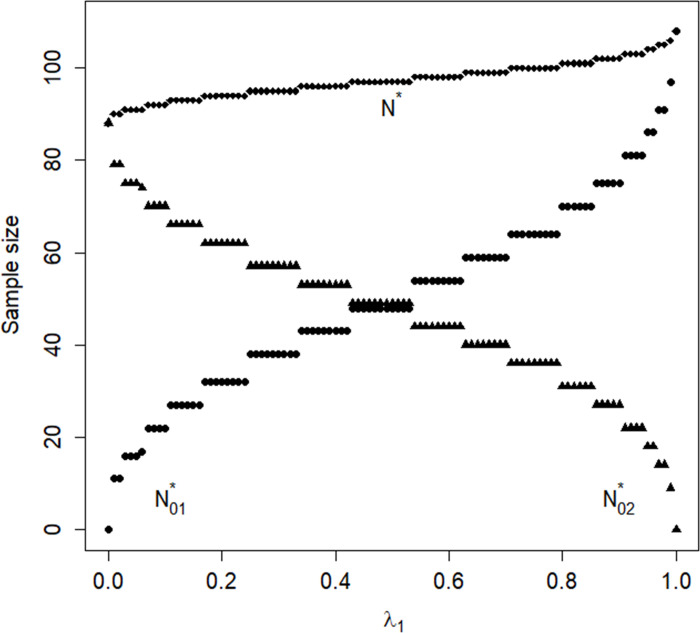
Optimal sample sizes as a function of the parameter *λ*_1_.

Fig 1 shows the optimal sample sizes $N_{01}^{*}$ for 10 minutes consultation time combined with 15 minutes and $N_{02}^{*}$ for 10 minutes consultation time combined with 20 minutes and the total sample size N* as a function of *λ*_1_ in a compound optimal design. The relation between *λ*_1_ and the two optimal sample sizes is not smooth since only natural values are taken into account. When *λ*_1_ = 0 then there is only interest in the comparison of 10 and 20 minutes consultation time. In that case, $N_{01}^{*}=0$ and $N_{02}^{*}=88$. When *λ*_1_ increases $N_{01}^{*}$ and consequently $N_{02}^{*}$ decreases. When *λ*_1_ = 1 then there is only interest in the comparison of 10 and 15 minutes consultation time. In that case $N_{01}^{*}=108$ and $N_{02}^{*}=0$. Furthermore Fig 1 shows that the total sample size increases from N* = 88 for *λ*_1_ = 0 to N* = 108 for *λ*_1_ = 1.

Suppose more interest lies in the comparison of 10 with 15 minutes than that of 10 minutes with 20 minutes. This goal may be achieved by choosing for instance *λ*_1_ = 0.75. The optimal sample sizes are then $N_{01}^{*}=64,N_{02}^{*}=36$, with total sample size N* = 100 and costs $N_{01}^{*}C_{s01}+N_{02}^{*}C_{s02}=99984.80$ euros. The optimal allocation assigns more general practitioners to the combination with 10 and 15 minutes consultation time than to the combination with 10 and 20 minutes. This is obvious since as there is more interest in the comparison of 10 minutes to 15 minutes than in the comparison of 10 minutes to 20 minutes, more general practitioners are assigned to that first combination of consultation times. On top of that, *C*_*s*01_ is larger than *C*_*s*02_ and the optimal allocation assigns more general practitioners to the combination with lower costs. The optimal allocation is more efficient than the uniform allocation, which has *N*_01_ = *N*_02_ = 48, but the uniform allocation is highly efficient with a relative efficiency of 0.923.

Given the optimal allocation, one can calculate which effects sizes can be detected for both contrasts at the available budget in a one-sided test with power level 0.8 and type I error rate 0.05. The effect size is calculated as $(z_{1-\alpha }+z_{1-\beta })\times \sqrt{var(\mu_{0}-\mu_{i})}$, where *z*_1−*α*_ and *z*_1−*β*_ are the 100(1−*α*)th and 100(1−*β*)th percentiles of the standard normal distribution. The variances of the contrasts are *var*(*μ*_0_−*μ*_1_) = 2.2 and *var*(*μ*_0_−*μ*_2_) = 4.2. The effect sizes are then calculated as 3.7 and 5.1 for the first and second contrast, respectively. This corresponds to standardized effect sizes as expressed by Cohen’s d of 0.35 and 0.46, respectively. So the minimum effects that can be detected in this trial are small (d = 0.2) to medium (d = 0.5) in size.

One can also calculate the power to detect a user-specified effect size for the optimal allocation. The power to detect a medium effect size is 0.97 for the first contrast and 0.86 for the second. Much lower power levels of 0.41 and 0.29 are achieved when a small effect size is to be detected.

## Discussion

The within-subject design is more efficient than the between-subject design. However, it is not always possible to evaluate the placebo and all treatments in each subject. In such a case the design is an incomplete-within subject design. This contribution studied optimal allocation of subjects to combinations of placebo with two treatments. The costs and variances were allowed to vary across placebo and treatments. Two optimality criteria that simultaneously take into account both placebo-treatment comparisons were considered and the design was sought under a budgetary constraint. In general it can be stated that more subjects are allocated to combinations of treatments that have high variance and low costs. For the compound optimal design the optimal allocation further depends on the weight *λ*_1_. When *λ*_1_ increases then there is more interest in the comparison of the placebo with treatment 1 rather than with treatment 2, and hence more subjects are allocated to combinations that include treatment 1.

Tables 1 and 2 give an overview of optimal allocations in a trial with a budget of 100000 for selected values of the costs and variances. Other combinations of the budget, costs and variances will, of course, result in different optimal allocations. A researcher who is planning to a trail should provide a priori estimates of the variances and covariances, along with the budget and costs and calculate the optimal allocation using the Shiny app. A priori estimates of the variances and covariances may be obtained from the literature or expert knowledge. Of course, there is no guarantee such estimates may hold in a different year, country or setting. It is therefore advised to do a robustness analysis. The optimal allocation may be derived for various plausible values of the variances and covariances. The allocation is robust if it hardly depends on these values. Otherwise, one may want to use robust optimal design techniques, such as an internal pilot or a maximin optimal design.

This contribution restricted to trials with one placebo and two treatments. In the incomplete-within-subject design three combinations could be distinguished: placebo with treatment 1, placebo with treatment 2 and treatment 1 with treatment 2. Of course it is possible to apply the optimal design methodology to trials with more than two treatments. It should be taken into account that more treatments imply more combinations of placebo and treatments and hence deriving the optimal allocation becomes more computationally demanding. Consider a trial with a placebo and three treatments. There are six combinations in the case two experimental conditions can be evaluated in each subject and another four combinations if three experimental conditions can be evaluated in each subject. These numbers reduce to three and three in the case the placebo should be evaluated in every subject. For four and more treatments the number of combinations even further increases. Finding the optimal allocation may then become very time-consuming.

The within-subject design is a longitudinal study design in which two or more measures on the same outcome are taken over time on each subject. Longitudinal studies are very often hampered by attrition, meaning that subjects leave the study prematurely. Attrition may occur when subjects lose interest in the study or experience severe side-effects from one treatment and are not willing to continue with the next treatment. Attrition results in a less efficient design by default and it should be avoided at all costs. However, this is often easier said than done in practice. As an alternative, one may derive optimal allocations that take into account anticipated rates of attrition. The number of subjects of such a trial is larger than in a trial without attrition so that the available budget is still efficiently used.

Another important direction for future research are optimal allocations in the case the outcome variable is categorical, such as the prevalence of some kind of behavior (i.e. binary outcome) or the severity of such behavior (i.e. ordinal outcome). It may also be of interest to study optimal allocations when subjects are nested within health professionals, such as clinical child psychologists in the first example of the introduction and sport coaches in the second example. Such health professionals may differ with respect to their skills, experience, empathy, motivation and enthusiasm, and such differences should be taken into account in the multilevel model by adding an extra level of nesting so that the between-professional variance can be estimated. In the design phase of such trials not only the number of subjects needs to be derived but also the number of health professionals.

In this paper the optimal allocation was found such that an optimality criterion was minimized for a given budget. In other words, the optimal design was based on efficiency. It is also possible to use another operating characteristic to derive the optimal allocation, namely the statistical power. Given user-specified values of the effect sizes and a desired power level, this means finding the optimal allocation such that either the costs or total sample size are minimized. The optimal allocation thus obtained may not necessarily be the same as the one based on efficiency. Future research may focus on optimal allocations based on statistical power, thereby building on existing work for the between-subject design with homogeneous costs and variances [19]. As multiple placebo-treatment comparisons are made, various characterizations of power can be made, depending on whether the hypothesis tests for these contrasts are considered individually of simultaneously. In the latter case the power is defined for rejecting at least one hypothesis or rejecting all hypotheses. It would be interesting to derive optimal allocations based on these various characterizations of power for the incomplete within-subject with heterogeneous costs and variances.

Thus far the literature on optimal allocations for placebo-treatment comparisons in the incomplete within-subject design was scarce. This contribution provides methodology along with a Shiny app for trials with a placebo and two treatments. I hope applied researchers will benefit from it and be able to derive optimal allocations for their trial so that they can compare placebo to treatments in an efficient way.
